# Mental health among first-generation college students: Findings from the national healthy minds study, 2018–2021

**DOI:** 10.1080/28324765.2023.2220358

**Published:** 2023-06-28

**Authors:** Sarah Ketchen Lipson, Yareliz Diaz, Jayne Davis, Daniel Eisenberg

**Affiliations:** a Department of Health Law Policy and Management, Boston University School of Public Health, Boston, USA; b Office of Scholarships and Student Aid, University of North Carolina, Chapel Hill, USA; c Department of Health Policy and Management, University of California Los Angeles Fielding School of Public Health, Los Angeles, USA

**Keywords:** Mental health, mental health treatment, college student populations, first-generation students

## Abstract

A mounting body of evidence reveals that college mental health outcomes are worsening over time. That said, little is known about the mental health needs of the nearly eight million first-generation students in U.S. postsecondary education. The present study uses population-level data from the national *Healthy Minds Study* to compare prevalence of mental health symptoms and use of services for first-generation and continuing-generation students from 2018–2021. The sample includes 192,202 students at 277 campuses, with 17.3% being first-generation. Findings reveal a high prevalence of mental health symptoms among both first-generation and continuing-generation students. Controlling for symptoms, FG students had significantly lower rates of mental health service use. Just 32.8% of first-generation students with symptoms received therapy in the past year, relative to 42.8% among continuing-generation students, and this disparity widened during the COVID-19 pandemic. Findings have important implications for the design and implementation of higher education policies, mental health delivery systems, college persistence and retention initiatives, and public health efforts in school settings.

## Introduction

In 2022, there were roughly 19 million undergraduate students enrolled in U.S. postsecondary education National Center for Education Statistics, (2022). A mounting body of evidence reveals that college student mental health outcomes are worsening over time; in the 2020–2021 academic year, over 60% of students were experiencing at least one clinically-significant mental health problem (based on validated screening tools), a roughly 50% increase since 2013 (Lipson et al., 2022). Despite a growing literature on mental health in college populations, little is known about the mental health needs of first-generation (FG) college students.

FG students are defined as “college students that are enrolled in postsecondary education whose parents do not have any postsecondary education experience” (Redford & Hoyer, 2017). About 40% of college attendees are FG students (Saenz et al., 2007), meaning there are nearly eight million FG students in U.S. higher education. While an important body of research focuses on the academic and basic needs of FG students, considerably less is known about the mental health of these students.

In addition to stressors that affect student populations writ large (e.g., adjusting to a college environment), FG students face unique stressors. On top of basic needs insecurity, many FG students also experience acculturative stress (Jenkins et al., 2013), as they navigate between home and school environments. Prior research has shown that FG students are more likely than continuing-generation (CG) students to experience post-traumatic stress, family achievement guilt, and to lack familial support (LeBouef & Dworkin, 2021; McFadden, 2016; Sy et al., 2011). Lower levels of familial support have been shown to be associated with stress and depression among FG students (Covarrubias et al., 2015). That said, much of the extant research on FG student wellbeing is limited by small sample sizes, single-site study designs, and a lack of validated measures of mental health.

The present study adds to the literature by using population-level data from the national *Healthy Minds Study* (HMS). Leveraging these large-scale data, we estimate the prevalence of key mental health outcomes as well as students’ use of mental health treatment, comparing FG and CG students at 277 U.S. colleges and universities. These data include years that span the COVID-19 pandemic, allowing us to examine potential changes during this time. This is the first known study of this scale to report on the mental health of FG students, and the findings have important implications for higher education policy, mental health delivery systems, and public health efforts in school settings.

## Materials and methods

### Data

Data come from the annual HMS, a national, cross-sectional survey examining mental health in college student populations (Healthy Minds Network, 2021). HMS was approved by institutional review boards on all participating campuses and covered by a National Institutes of Health Certificate of Confidentiality. The present study analyzes six semesters of HMS data (fall 2018-spring 2021), which include students from 277 U.S. colleges and universities. In addition to representing both community colleges and four-year institutions, the study sites are diverse across numerous campus characteristics, including enrollment size, public vs. private, and geographic region. The study design and protocol of HMS has been documented extensively in prior publications (Goodwill & Zhou, 2020; Lipson & Eisenberg, 2017; Lipson et al., 2022). At participating campuses with > 4,000 students, a random sample of 4,000 degree-seeking students from the full population was invited to HMS; at smaller institutions, all students were invited. The only exclusion criterion was that students had to be at least 18 years of age. HMS is a mobile survey, completed by students on computers, smart phones, or tablets. Recruitment is conducted by email. Upon clicking a personalized link in the recruitment email, students were presented with an informed consent page and had to agree to the terms of participation before entering the survey. Data were collected using Qualtrics software. To incentivize participation, students were informed of their eligibility for one of 12 cash prizes totaling $2,000 annually (two $500 and 10 $100 gift cards). Response rates were 16% in 2018–2019, 16% in fall 2019, 13% in winter/spring 2020, 14% in fall 2020, and 15% in winter/spring 2021. To adjust for potential differences between responders and non-responders, the study team constructed sample probability weights. Administrative data were obtained from participating institutions, including sex, race/ethnicity, and grade point average. These data were used to construct response weights, equal to 1 divided by the estimated probability of response. Thus, weights are larger for respondents with underrepresented characteristics, ensuring that estimates are representative of the full population in terms of these known characteristics.

### Key measures

First-generation status: First-generation status is based on parental education as reported by students in HMS. Students were asked “What is the highest level of education completed by your parents or stepparents?” and were asked this question twice, allowing students to report educational attainment for up to two parental figures. Response options were: “8th grade or lower”, “Between 9th and 12th grade (but no high school degree)”, “High school degree”, “Some college (but no college degree)”, “Associate’s degree”, “Bachelor’s degree”, and “Graduate degree”. Students were categorized as being FG if the highest level of parental education (for all parental figure(s) reported on) was “8th grade or lower”, “Between 9th and 12th grade (but no high school degree)”, or “High school degree”. Students were categorized as CG if the highest level of parental education for one or more parent was “Some college (but no college degree)”, “Associate’s degree”, “Bachelor’s degree”, or “Graduate degree”.

Mental health status: We examine four outcomes related to mental health symptom prevalence; binary outcomes are used because most have been validated based on standard cutoffs and reported in prior studies Lipson et al., 2022). For outcomes derived from continuous scales (flourishing, depression, and anxiety symptoms) we also report mean scores in the results table. (1) To estimate the proportion of students who are flourishing, we use the eight-item Flourishing Scale (Diener et al., 2010). Scores range from 8 to 56, with higher scores indicating higher wellbeing. This scale does not have a recommended cutoff; rather a score of > 48 was selected because it best matches rates of flourishing from other scales (e.g., the Mental Health Continuum (Keyes, 2002)) in college populations, and has been used widely in prior HMS publications. (2) Symptoms of depression are examined using the Patient Health Questionnaire-9 (PHQ-9) (Lowe et al., 2004). Across settings and populations, the PHQ-9 has been validated as internally consistent (Huang et al., 2006). Scores range from 0 to 27; the standard cutoff of ≥ 10 is used. (3) Symptoms of anxiety are measured by the Generalized Anxiety Disorder 7-item scale (GAD-7) (Spitzer et al., 2006). Scores range from 0–21; the standard cutoff of ≥ 10 is used, which has been shown to have high sensitivity (89%) and specificity (82%) (Spitzer et al., 2006). (4) A single question, originally developed for the National Comorbidity Survey (Kessler et al., 2005), is used to assess suicidal ideation: “In the past year, did you ever seriously think about attempting suicide?” Responses are “yes” and “no”.

**Table 1.** Sample characteristics

	All students (*N*=192,202)	FG students (*N*=27,738)	CG students (*N*=164,464)
**Financial stress**			
Always/often stressful	40.56%	54.17%	37.71%
Sometimes/rarely/never stressful	59.44%	45.83%	62.29%
**Age**			
18–21	69.35%	53.18%	72.74%
22–25	16.63%	17.49%	16.45%
26–30	5.50%	9.28%	4.70%
31+	8.52%	20.04%	6.11%
**Gender identity**			
Cisgender man	40.97%	36.76%	41.85%
Cisgender woman	55.86%	61.13%	54.76%
TNB	3.17%	2.11%	3.39%
**Race/ethnicity**			
AI/AN	0.34%	0.48%	0.31%
Arab Am	0.87%	1.16%	0.81%
APIDA	5.93%	7.39%	5.63%
Black	10.62%	15.65%	9.57%
Latinx	8.21%	25.20%	4.66%
Multiracial	9.72%	8.71%	9.93%
White	64.30%	41.41%	69.08%

Notes: Table values are weighted percentages. “FG” is “first-generation”; “CG” is “continuing-generation”; “TNB” is “transgender and nonbinary”; “AI/AN” is “American Indian/Alaskan Native”; “Arab Am” is “Arab American”; “APIDA” is “Asian/Pacific Islander/Desi American”.

Treatment: We examine three outcomes related to past-year mental health treatment: (1) any treatment (therapy and/or medication), (2) therapy, and (3) psychotropic medication use. In order to understand variations not attributed to differences in clinical need, we examine these outcomes among students meeting criteria for depression, anxiety, and/or suicidal ideation (as defined above). As a secondary analysis, we examine barriers to mental health treatment among students with symptoms. Students were asked why they had not received treatment or received fewer services than they otherwise would have, and were instructed to “select all that apply” from the following list: “I haven’t had the chance to go but I plan to”, “No need for services”, “Financial reasons”, “Not enough time”, “Not sure where to go”, “Difficulty finding an available appointment”, “Prefer to deal with issues on my own or with support from family/friends”, and “Other”.

### Statistical analysis

Analyses are descriptive in nature, intended to document variations in mental health prevalence and service utilization outcomes by FG student status. For mental health status measures, we report the weighted percentage of FG and CG students meeting criteria for flourishing, depression, anxiety, and suicidal ideation from 2018–2021. We also report means and standard errors for flourishing, depression, and anxiety scores; we calculate effect sizes for these continuous outcomes (difference in means divided by standard deviation). We estimate logistic regression models for each of these outcomes, controlling for financial stress, age, gender identity, and race/ethnicity, reporting adjusted odds ratios (aOR), 95% confidence intervals (CI), and statistical significance; CG is the reference group. Financial stress, a covariate in the regression models, is based on a survey item that asked students: “How would you describe your financial situation right now?” Response options were “Always stressful”, “Often stressful”, “Sometimes stressful”, “Rarely stressful”, and “Never stressful”. Students who reported “Always stressful” or “Often stressful” were categorized as having financial stress. To examine potential changes during the COVID-19 pandemic, we repeat these analyses—weighted percentages, means, and logistic regressions—using just the 2020–2021 academic year. Next, we report the percentage of FG and CG students with past-year treatment, therapy, and medication among those with a positive screen for depression, anxiety and/or suicidal ideation. We discuss the mental health “treatment gap” (Kohn et al., 2004), defined as the proportion of students with symptoms who are not receiving treatment. Our focus on the treatment gap is not meant to imply that all students meeting criteria for one or more mental health problems necessarily require treatment; rather the treatment gap is meant to help quantify levels of unmet need accounting for symptom prevalence. With the sample restricted to those with symptoms, we estimate logistic regression models for the service utilization outcomes, again controlling for financial stress, age, gender identity, and race/ethnicity, reporting aOR, 95% CI, and *p*-values with CG as the reference. Given the large sample size, we highlight only those aORs from the regression models with statistical significance at *p* < .001. We repeat analyses using just the 2020–2021 data. To measure the extent to which the institutional level accounted for overall variance in outcomes, we calculated intraclass correlation coefficients, finding small but statistically significant correlations: 0.01 (*p* < .001) for depression, 0.01 (*p* < .001) for anxiety, 0.005 (*p* < .001) for suicidal ideation, and 0.02 (*p* < .001) for treatment utilization. This indicates that campus-level variation is small when compared with overall variation in mental health and help-seeking. Barriers to treatment (as reported by students with untreated symptoms) are presented separately for FG and CG students. All analyses were conducted using Stata 17 and weighted using the sample weights described.

## Results

### Sample characteristics

The analytic sample is restricted to U.S. undergraduates, defined as students in associate’s or bachelor’s programs. The sample includes 192,202 students from 277 campuses, with over 25,000 FG students (17.3% of the overall sample). More than half of FG students (54.2%) reported their financial situation as always/often stressful relative to 37.7% of CG students. Overall, 64.3% of the analytic sample identified as White. A lower proportion of FG students identified as White (41.4%) relative to CG students (69.1%). More than one-in-four FG students identified as Latinx (25.2%) relative to just 4.7% of the CG sample. A higher proportion of CG students (72.7%) were between the ages of 18 and 21, relative to FG students (53.2%). In the overall sample, 41% identified as cisgender male, 55.9% as cisgender female, and 3.2% as transgender or nonbinary. A higher proportion of FG students identified as cisgender women (61.1%) relative to CG students (54.8%). Full sample characteristics are presented in Table 1.

### Mental health status

As presented in Table 2, mental health problems are highly prevalent in the overall sample. Relative to CG students, FG students had slightly lower levels of flourishing (36.2% vs. 37.0%) and slightly higher levels of depression (43.1% vs. 40.4%) and anxiety (36.4% vs. 34.1%), but these differences were not statistically significant when controlling for covariates in the adjusted logistic regression models. When comparing differences in means for continuous outcomes, effect sizes were all small. Both groups reported high rates of suicidal ideation, with CG students having slightly higher prevalence (15.1%) relative to FG students (14.8%).

**Table 2.** Variations in mental health prevalence and service utilization by first-generation student status

		All years					2020–2021			
	FG	CG	ES	aOR (95% CI)	*p*	FG	CG	ES	aOR (95% CI)	*p*
**Mental health status**										
Flourishing (≥48)	36.16%(*m*=42.67, SE=.099)	37.01%(*m*=43.15, SE=.036)	−.05	.96 (.91, 1.01)	.102	36.03%(*m*=42.43, SE=.15)	36.03%(*m*=42.86, SE=.05)	−.05	.98 (.91, 1.05)	.528
Depression (PHQ-9≥10)	43.14%(*m*=9.40, SE=.072)	40.35%(*m*=9.05, SE=.027)	.05	1.02 (.97, 1.08)	.359	44.19%(*m*=9.63, SE=.103)	42.77%(*m*=9.43, SE=.04)	.02	.99 (.93, 1.06)	.863
Anxiety (GAD-7≥10)	36.37%(*m*=7.95, SE=.07)	34.10%(*m*=7.69, SE=.023)	.04	1.05 (1.0, 1.10)	.06	36.73%(*m*=8.09, SE=.091)	36.08%(*m*=8.01, SE=.035)	.01	1.02 (.95, 1.09)	.583
SI, past year	14.75%	15.05%		.99 (.93, 1.06)	.853	13.74%	14.62%		.96 (.88, 1.05)	.382
**Service utilization**										
Any treatment	45.48%	55.08%		.73 (.68, .78)	<.001	42.70%	54.27%		.67 (.62, .74)	<.001
Counseling/therapy	32.82%	42.84%		.70 (.66, .75)	<.001	30.41%	41.76%		.66 (.60, .72)	<.001
Psychotropic med	29.65%	36.82%		.77 (.72, .83)	<.001	27.94%	36.53%		.73 (.67, .81)	<.001

Notes: ‘FG’ is ‘first-generation’; ‘CG’ is ‘continuing-generation’; ‘SI’ is ‘suicidal ideation’. Tables values are weighted percentages, mean scores (‘m’) with standard errors (SE) and effect sizes (ES) (for flourishing, depression, and anxiety), and adjusted odds ratios (aOR) from logistic regressions with 95% confidence intervals (CI), and p-values, controlling for age, gender identity, financial stress, and race/ethnicity. CG is the reference group. Past-year service utilization outcomes are examined among students with a positive screen for depression, anxiety, and/or SI.

### Mental health treatment

As presented in Table 2, among FG students with depression, anxiety and/or suicidal ideation, 45.5% had received any mental health treatment in the past year (a 54.5% treatment gap) relative to 55.1% of CG students (a 44.9% treatment gap). This disparity in service use was apparent both for counseling/therapy and psychotropic medication use. Less than one-third of FG students with symptoms (32.8%) received counseling/therapy in the past year relative to 42.8% of CG students. Similarly, just 29.7% of FG students with symptoms used psychotropic medication compared to 36.8% of CG students. In adjusted logistic regression models, FG students had 0.73 lower odds of receiving treatment (*p* < .001), 0.70 lower odds of counseling/therapy (*p* < .001), and 0.77 lower odds of medication use (*p* < .001).

**Figure 1.**
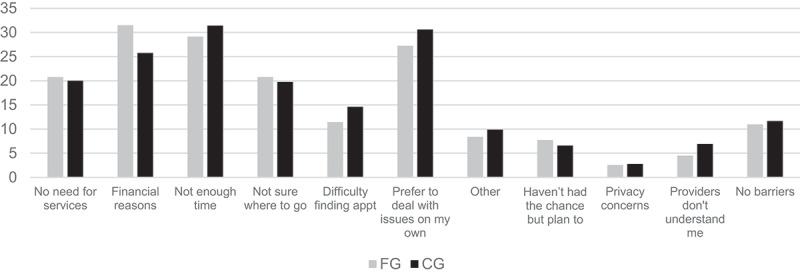
Barriers to mental health treatment among students with symptoms.

Barriers to mental health treatment as reported by students with a positive screen for depression/anxiety and/or reporting suicidal ideation reveal similar patterns for FG and CG students with one exception (Figure 1). A higher proportion of FG students reported financial reasons as a salient barrier to treatment (31.5%) relative to CG students (25.8%).

Figure values are weighted percentages among students with a positive screen for depression, positive screen for anxiety, and/or past-year suicidal ideation. “FG” is “first-generation”; “CG” is “continuing-generation”; “appt” is “appointment”.

### Trends pre- vs. during-pandemic

Looking at trends during the 2020–2021 academic year, data reveal slightly lower levels of flourishing and higher levels of depression and anxiety than prior to the pandemic; this is true for both FG and CG students. Differences between FG and CG students were largely consistent with the above-noted differences during all years of data examined, with FG students continuing to have lower rates of service utilization relative to CG students. The most notable shift was for past-year treatment among students with a positive screen; during the pandemic, the “treatment gap” widened between FG and CG students, with CG student help-seeking remaining consistent with prior years and FG student help-seeking falling lower than pre-pandemic. In 2020–2021, just 42.7% of FG students with clinically-significant symptoms received any mental health treatment in the past year (vs. 45.6% in all years) compared to 54.3% of CG students (vs. 55.1% in all years). This pattern held true for both counseling/therapy and psychotropic medication use, with FG having significantly lower odds of receiving care in 2020–2021 when controlling for covariates.

## Discussion

Very little is known about the state of mental health among the roughly eight million first-generation students in U.S. higher education. To fill this gap, the present study used national data from the *Healthy Minds Study* to compare the prevalence of symptoms and treatment use among FG and CG students enrolled at 277 U.S. colleges and universities from 2018 to 2021.

### Understanding and addressing inequalities in mental health prevalence

We find that both FG and CG students had high levels of prevalence for depression, anxiety, and suicidality. More than 40% of students in each group screened positive for symptoms of depression and over one-third for anxiety. Levels were slightly higher among FG students, but there were no significant differences in prevalence. Roughly 15% of both groups reported seriously considering suicide in the past year.

Prior research has revealed that financial stress and debt are key risk factors for suicide (Gertner et al., 2019). Though income was not measured in the present study, approximately half of all FG students in the U.S. are also low-income (Saenz et al., 2007), meaning that financial stress affects a large proportion of FG students. In addition to the urgent need for federal policy change, our study underscores opportunities within higher education to reduce these risk factors. Efforts to reduce basic needs insecurity (e.g., campus food pantries) can—and in fact must—be thought of as part of a public health approach to addressing student mental health and reducing inequalities. This assertion implicates a much broader set of institutional stakeholders, most existing outside of a campus health system or specific programs for FG students. For example, policies around financial aid, housing, and dining must be considered from the perspective of advancing mental health equity and reducing risk factors. There are numerous, specific opportunities and these will vary across states as well as the diversity of institutional settings and student needs, but some include: investing in financial aid Promise Programs allowing students to graduate from college with little or no debt; providing housing for students that cannot go home during breaks; having a simple way for unused meal swipes/dining points to be shared with students facing food insecurity; offering peer mentoring programs to help FG students navigate college and foster connection with other students; and equipping (and reminding) faculty/staff to explain the jargon and procedures of higher education (e.g., offices hours, institutional resources) and understand the importance of discussing campus resources appropriate for students’ presenting needs/concerns, where they are located, how to access them, and what to expect from the resource.

### Understanding and addressing inequalities in mental health service use

In terms of mental health service utilization, our data show that FG students had lower rates of accessing treatment, especially therapy. There is an overarching need to invest in mental health prevention efforts and screenings that reach FG students. This need is more urgent than ever, given that during the pandemic, the mental health “treatment gap” widened between FG and CG students. When it comes to narrowing the “treatment gap” for FG students, there is a powerful economic case for institutions to invest in mental health resources. Our team’s prior research with HMS has found that untreated depression is associated with a two-fold increase in the likelihood of dropping out or stopping out of college without graduating, even when controlling for prior academic performance and test scores (Eisenberg et al., 2009). Given that FG students are less likely to access mental health treatment and also more likely to drop/stop out of college, there will be a strong return on investment for expanding the reach of mental health services to meet the unique needs of FG students. Having culturally relevant and accessible services available (whether at times conducive to working students, in languages other than English, and/or without cost) is not a complete solution on its own, further efforts are needed to connect students to available mental health resources and to motivate service uptake. There is also a need for grants and donors to provide funding to cover outside therapy when students need therapy/counseling that extends beyond institutional services offered. Academic units (departments, chairs, faculty, advisors, etc.) have an important role to play in addressing the inequalities revealed in the present study. Research has shown that FG students are less likely to access other types of campus resources as well, whether academic, extracurricular, or career-related (Stebleton & Soria, 2012). Advisors are uniquely positioned to remind students that prioritizing their mental health—though it may seem like a competing demand—is likely to benefit their academic performance. This type of messaging is consistent with best practices from *Motivational Interviewing* (Miller & Rollnick, 2013), the goal being to align a desired behavior, in this case prioritization of mental health (or more broadly, utilization of supportive services and resources)—whether through health behavior change (e.g., sleep, exercise, substance use) or use of clinical services—with students’ goals. Many students are unaware of how their mental health relates to their academic performance, and faculty can remind students how their mental health is inextricably linked to their scholarly pursuits. This messaging may be especially effective when delivered repeatedly, beginning with incoming undergraduates starting their college experience.

Given that FG students are less likely to receive services, there is an urgent need for population-level approaches to reach FG students. That said, there are also implications for clinicians working with FG students, ensuring that treatment goals accurately reflect the needs of students. Mental health providers should endeavor to understand FG students’ priorities, the obstacles they have overcome to seek help, and take a strengths-based approach with new FG student clients; this includes looking for hidden strengths such as flexibility, code switching, and decision-making. In order to support FG students, clinicians also need to build an understanding of the unique challenges that many FG students face, including pressure to financially support family beginning in college. It is important for clinicians to build an alliance with FG students—getting to know them and their story and listening for hope and resilience.

### Limitations

Leveraging large-scale, national data, we examined mental health needs among FG students, a population that has often been omitted from prior studies on college mental health. The study incorporated validated screening tools to measure symptom prevalence, with consistent measures across years. Generalizability of findings is strengthened by the multisite nature of HMS (with 277 campuses included) as well as random sampling at the student level. In addition to these strengths, there are several limitations to consider. First, annual response rates ranged from 13% to 16%; though this is typical for online surveys (Duffy et al., 2019; Lipson et al., 2018), it clearly raises the potential of nonresponse bias. The researchers applied nonresponse weights along known characteristics, but there may be differences on unobserved characteristics. Second, campuses elected to participate in HMS; though the institutional sample is large and diverse, it is not random, and the institutional sample differs each year. Importantly, prior research with HMS has consistently found—through estimations of random-effects regression models and ICCs—that campus-level variation is small compared to individual-level variation in student mental health and help-seeking (Lipson et al., 2015); as noted, this was true in the present data. Finally, although mental health outcomes were measured with validated screens, it is important to remember that these measures do not represent clinical diagnoses.

## Conclusions

This is the first known study of this scale to report on the mental health of first-generation college students. The data reveal high prevalence of mental health problems and significant disparities in the use of services relative to continuing-generation students. These findings have important implications for higher education policy, mental health delivery systems, and public health prevention efforts in school settings. Further research is needed in this area, including examination across intersectional identities, with measures of low-income status, and with longitudinal data.

## Data Availability

Data from the Healthy Minds Study (HMS) are made publicly available (with precautions to protect respondent and institutional anonymity). HMS data requests can be made online at the following link: https://docs.google.com/forms/d/e/1FAIpQLSdG96RypTFpxqOVAtkECv7Rbd7q5taoLi7Rq-BKpCWdkM6DmQ/viewform.
